# Thyroid function and anemia: results from the Brazilian Longitudinal Study of Adult Health

**DOI:** 10.1590/1806-9282.20260490

**Published:** 2026-09-28

**Authors:** Alinne de Santo Nascimento, Marcos Rafael Nogueira Cavalcante, Angelita Gomes de Souza, Isabela Judith Martins Benseñor

**Affiliations:** 1 Universidade de São Paulo, Hospital Universitário, Centro de Pesquisa Clínica e Epidemiológica – São Paulo (SP), Brazil.

**Keywords:** Thyroid hormones, Thyroid stimulating hormone (TSH), Anemia

## Abstract

**OBJECTIVE:**

The aim of this study was to analyze the association of thyroid stimulating hormone and free-thyroxin with anemia as well as the role of several sociodemographic variables in these associations.

**METHODS:**

This is a cross-sectional analysis of the Brazilian Longitudinal Study of Adult Health study (2008–2010), a prospective cohort study of civil servants in six locations in Brazil. Inclusion criteria: to be an active or retired civil servant of one of the institutions and 35–74 years at enrollment. Exclusion criteria: pregnancy less than 4 months before entering the study for women, intention to quit the institution soon, live outside the metropolitan region where the investigation center is located or communication problems. Anemia was defined as hemoglobin <12 g% in women and <13 g% in men. After exclusions, 13,247 participants were included in the analysis. Models were presented without adjustment and adjusted for sociodemographic and clinical risk factors. The study was approved by the Ethic Committee.

**RESULTS:**

Median age was 51.9 (51–53 years), 54% of women There was an association of less presence of anemia with higher free-thyroxin levels considering the entire sample: OR 0.80 (95%CI 0.65–0.98) for the second quintile; OR 0.55 (95%CI 0.43–0.70) for third quintile; OR 0.49 (95%CI 0.38–0.63) for fourth quintile; and OR 0.52 (95%CI 0.40–0.66) for fifth quintile for free-thyroxin levels. This association was also present when analyzing the sample by sex.

**CONCLUSION:**

Higher levels of free-thyroxin are associated with lower odds of anemia. No associations were found between thyroid stimulating hormone levels and anemia. In analysis, according to sex, No differences were found between men and women.

## INTRODUCTION

Thyroid diseases are very prevalent worldwide with an age-standardized prevalence around 5%^1^. In Brazil, prevalence of subclinical and overt hypothyroidism were respectively 5.4 and 7.4%^2^. Anemia is also a prevalent disease, impacting 27% of the world's population^3^. Some studies reported an association of clinical and subclinical hypothyroidism with anemia, as patients with hypothyroidism may present low antioxidant potential, leading to an increase in inflammatory cytokines that induce alterations, potentially interfering with different erythropoiesis pathways, which could lead to anemia^3–5^.

Some studies evaluated the association of thyroid diseases and anemia. In 2018, a meta-analysis conducted as part of the Thyroid Studies Collaboration investigated the associations between thyroid dysfunction and anemia. The findings showed that levels of hemoglobin were lower across all thyroid dysfunction subgroups compared to euthyroid participants^6^. In the cross-sectional study of Vliet et al^7^, including 445,482 participants, the authors did not report any association between thyroid stimulating hormone (TSH) or free-thyroxin (FT4) levels with anemia. In another cross-sectional analysis (n=12,337), the adjusted Hb was 0.22 g/dL lower in overt hypothyroidism compared to euthyroid participants^4^. In a prospective analysis, patients with subclinical and overt hypothyroidism at baseline showed a trend of increased risk of developing anemia during follow-up^8^. Puy et al conducted a study to assess whether levothyroxine treatment leads to a rise in hemoglobin levels in patients with subclinical hypothyroidism. The change in hemoglobin levels was not significantly different between the levothyroxine and the placebo groups^9^.

In this study, we aim to analyze the association of TSH and FT4 with anemia using data from the Brazilian Longitudinal Study of Adult Health (ELSA-Brasil), as well as the role of several sociodemographic variables in this association.

## METHODS

This is a cross-sectional analysis using data about thyroid function and hemoglobin levels from ELSA-Brasil baseline (2008–2010), a cohort study following 15,105 civil servants aged 35–74 years from six institutions (Federal Universities of Minas Gerais, Espírito Santo, Rio Grande do Sul and Bahia, Oswaldo Cruz Foundation and University of São Paulo), with the objective of investigating the incidence of cardiovascular disease, diabetes and other chronic diseases. In this analysis we used data from the baseline examination (2008–2010)^10^. Inclusion criteria of the study were to be an active or retired civil servant of one of the institutions and 35–74 years at enrollment. Exclusion criteria were pregnancy less than 4 months before entering the study for women, intention to quit the institution soon, live outside the metropolitan region where the investigation center is located or communication problems. More information about the study is elsewhere^10–12^.

### Study population

Of 15,105 participants included in the baseline of ELSA-Brasil, 53 presented missing data on anemia, 1,604 presented missing data on thyroid function and 254 for using medication that interfered with the thyroid function, totaling after exclusions a sample of 13,247.

### Thyroid function

TSH and FT4 were determined by a third-generation immunoenzymatic assay (Roche Diagnostic, Manheim, Germany) at baseline. The analysis included euthyroid participants (TSH 0.4–4.0 mIU/L) with no use of levothyroxine or antithyroid medication and participants with subclinical hypothyroidism (TSH>4.0 mIU/L, FT4 within reference levels) and overt hypothyroidism (TSH>4.0 mIU/L, low FT4). TSH reference range was 0.4–4.0 mlU/L, FT4 reference range was 0.93–1.7 ng/dL.

### Anemia

Blood cell counts were performed with automated equipment and anemia was defined in women if Hb<12 g/dL and in men if Hb<13 g/dL.

### Other variables

Sociodemographic variables were categorized as: age, included as a continuous variable; race, classified according to the Brazilian National Census as White, Mixed (pardo), Black, Asian, and indigenous; Education was categorized as less than high-school, high school and incomplete college or complete college or more. Average familiar monthly net income was classified as: <1,245, 1,245–3,319 and ≥3,320 (1US$=2R$ 2.00 at baseline). Comorbidities are defined as the presence of at least one hypertension, diabetes, dyslipidemia, depression, anxiety, obesity, coronary heart diseases, and stroke. It was categorized as no comorbidity, 1 comorbidity and two or more comorbidities.

### Statistical analysis

Categorical variables were expressed as counts and percentages and compared using the chi-squared test. The Shapiro-Wilk test was applied to the age, TSH, and FT4 values to assess the assumption of normality. As they presented a non-normal distribution, we used the Kruskal-Wallis test to compare continuous variables.

TSH and FT4 were divided into quintiles. We used logistic regression models expressed as odds ratio (OR) and their respective 95%CIs presented in models without adjustment and multivariable adjusted for both sociodemographic and chronic conditions (Model 1): for age, sex, race, education, hypertension, and diabetes. These analyses were performed with "R" software version 4.4.2 and R Studio 2024.04.2 Build 764.

## RESULTS

Table 1 shows general characteristics of the sample according to FT4 quintiles. Mean hemoglobin levels were 13.8 (1.4) for the first quintile; 14.0 (1.3) for the second quintile; 14.2 (1.3) for the third quintile; 14.3 (1.3) for the fourth quintile; 14.4 (1.3) for the fifth quintile (p<0.0001). Table 2 shows the general characteristic of the sample according to TSH quintiles.

**Table 1:** General characteristics of the sample according to free-thyroxin quintiles.

General characteristics (ng/dL)		0.02–1.04 (2,662)	1.05–1.14 (2,910)	1.15–1.22 (2,606)	1.23–1.32 (2,448)	1.33–5.80 (2,621)	p-value
Anemia (%)		217 (8.1)	187 (6.4)	111 (4.2)	90 (3.6)	98 (3.7)	<0.0001
Hemoglobin levels (g/dL)		13.7 (12.9–14.7)	13.9 (13.1–15.0)	14.1 (13.3–15.1)	14.3 (13.4–15.2)	14.4 (13.5–15.3)	<0.0001
Woman (%)		1,700 (63.8)	1,673 (57.4)	1,368 (52.4)	1,216 (49.6)	1,258 (48.0)	<0.0001
Age median (1Q–3Q)		51 (45.0–58.0)	51 (45.0–58.0)	51 (45.0–58.0)	51 (45.0–58.0)	52 (45.0–59.0)	0.0025
Race (%)							
	White	1,351 (51.2)	1,443 (50.0)	1,373 (53.4)	1,305 (54.0)	1,433 (55.4)	<0.0001
	Mixed	772 (29.2)	848 (29.4)	682 (26.5)	655 (27.1)	659 (25.5)	
	Black	442 (16.7)	502 (17.4)	423 (16.4)	362 (15.0)	353 (13.6)	
	Asian	42 (1.5)	63 (2.1)	65 (2.5)	68 (2.8)	103 (3.9)	
	Indigenous	28 (1.0)	28 (0.9)	26 (1.0)	23 (0.9)	36 (1.3)	
Education (%)							
	<High-school	342 (12.8)	363 (12.4)	306 (11.7)	317 (12.9)	359 (13.6)	<0.0001
	High school/some college	1,004 (37.7)	1,106 (38.0)	895 (32.8)	805 (32.8)	829 (31.6)	
	College or more	1,316 (49.4)	1,441 (49.5)	1,405 (53.9)	1,326 (54.1)	1,433 (54.6)	
Income (US$)							
	Up to 1,245	764 (28.8)	785 (27.0)	643 (24.8)	638 (26.1)	632 (24.1)	0.0003
	1,245–3,319	1,174 (44.3)	1,299 (44.8)	1,153 (44.5)	1,063 (43.6)	1,152 (44.0)	
	≥3,320	710 (26.8)	814 (28.0)	795 (30.6)	737 (30.2)	829 (31.7)	
	Hypertension (%)	875 (32.9)	978 (33.6)	914 (35.0)	845 (34.5)	1,043 (39.8)	<0.0001
	Diabetes (%)	387 (14.5)	424 (14.5)	398 (15.2)	399 (16.3)	464 (17.7)	0.0059
	Dyslipidemia (%)	1,263 (47.6)	1,308 (45.0)	1,174 (45.2)	1,085 (44.3)	1,240 (47.4)	0.0552
	(BMI kg/m^2^) median (1Q–3Q)	27.2 (24.3–30.5)	26.5 (23.9–29.8)	26.2 (23.4–29.4)	26.0 (23.5–28.8)	25.8 (23.3–28.9)	<0.0001
Comorbidities							
	0	626 (23.5)	735 (25.2)	682 (26.1)	722 (29.4)	632 (24.1)	<0.0001
	1	890 (33.4)	1,004 (34.5)	874 (33.5)	768 (31.3)	900 (34.3)	
	2 or more	1,146 (43.0)	1,171 (40.2)	1,050 (40.2)	958 (39.1)	1,089 (41.5)	

Anemia was defined as <13 g/dL for men and <12 g/dL for women. Comorbidities have been included as the presence of: HAS, diabetes, dyslipidemia, depression, anxiety, obesity, coronary heart disease (HMPA10 and HMPA45), and stroke.

**Table 2:** Comparison between euthyroid and subclinical hypothyroidism samples.

General characteristics		Euthyroid	Subclinical hypothyroidism	p-value
Anemia (%)		572 (5.2)	43 (3.8)	0.0538
Hemoglobin levels (g/dL)		14.1 (13.2–15.1)	14.2 (13.3–15.2)	0.1011
Women (%)		5,637 (51.8)	589 (53.3)	0.4722
Age, years median (1Q–3Q)		51 (45–58)	53 (47–60)	<0.0001
Self-reported race (%)				
	White	5,475 (50.9)	667 (60.6)	<0.0001
	Mixed	3,034 (28.2)	281 (25.5)	
	Black	1,842 (17.1)	107 (9.7)	
	Asian	280 (2.6)	28 (2.5)	
	Indigenous	114 (1.0)	16 (1.4)	
Education attainment (%)				
	Less than high-school	1,399 (12.8)	156 (14.0)	0.3287
	High school and some college	3,879 (35.6)	375 (33.7)	
	Complete college or more	5,597 (51.4)	580 (52.2)	
Income (US$)				
	Up to 1,245	4,756 (43.9)	530 (47.8)	0.0171
	1,245–3,319	3,140 (28.9)	315 (28.4)	
	≥3,320	2,937 (27.1)	262 (23.6)	
Smoking (%)				
	Never	6,138 (56.4)	656 (59.0)	<0.0001
	Past	3,206 (29.4)	358 (32.2)	
	Current	1,530 (14.0)	97 (8.7)	
Alcohol intake (%)				
	Never	1,124 (10.3)	122 (11.0)	0.7825
	Past	2,124 (19.5)	213 (19.2)	
	Current	7,617 (70.1)	774 (69.7)	
	Single (%)	3,572 (32.8)	395 (35.5)	0.0729
	Hypertension (%)	3,792 (34.8)	410 (36.9)	0.188
	Diabetes (%)	1,692 (15.5)	167 (15.0)	0.6824
	Dyslipidemia (%)	4,912 (45.1)	505 (45.4)	0.8881
	Body-mass index (BMI kg/m^2^) median (1Q–3Q)	26.2 (23.6–29.4)	26.6 (23.9–29.6)	0.0675
Comorbidities				
	0	2,872 (26.4)	285 (25.6)	0.6467
	1	3,601 (33.1)	383 (34.4)	
	2 or more	4,402 (40.4)	443 (39.8)	

Anemia was defined as <13 g/dL for men and <12 g/dL for women; Model 1 includes age, race, education attainment, and income; Model 2 includes age, race, education attainment, diabetes mellitus, and hypertension. BMI: body mass index.

Frequency of anemia decreased according to FT4 quintiles. The lower FT4 quintile presented the higher frequency of women (p<0.0001). Frequency of White, Asian and Indigenous participants increased according to FT4 quintiles, while frequency of Mixed and Black participants decreased (p<0.0001). Frequency of College education (p<0.0001) was higher in participants of the first quintile. Frequency of hypertension (p<0.0001) was higher in participants of the fifth quintile. Although differences in body mass index (BMI) were statistically significant according to FT4 levels, those differences were clinically irrelevant.

Table 3 shows the association of anemia with TSH and FT4 in the sample according to sex and the entire sample. Using the first quintile as reference, there was an association of less presence of anemia with higher FT4 levels present in males, and also in females. Such association was also present when considering the entire sample.

**Table 3:** Association of thyroid stimulating hormone levels and free-thyroxin with presence of anemia, presented according to men, women, and the entire sample.

		TSH		FT4
Male	Crude model	Q1	1 (Reference)	1 (Reference)
		Q2	0.66 (0.39–1.09)	0.51 (0.33–0.78)
		Q3	0.76 (0.47–1.24)	0.41 (0.26–0.64)
		Q4	0.96 (0.60–1.53)	0.34 (0.21–0.55)
		Q5	1.07 (0.68–1.70)	0.31 (0.19–0.49)
	Model 1	Q1	1 (Reference)	1 (Reference)
		Q2	0.66 (0.39–1.11)	0.52 (0.33–0.79)
		Q3	0.79 (0.48–1.30)	0.43 (0.27–0.68)
		Q4	1.04 (0.65–1.67)	0.38 (0.23–0.62)
		Q5	1.14 (0.71–1.83)	0.31 (0.19–0.51)
Female	Crude model	Q1	1 (Reference)	1 (Reference)
		Q2	0.80 (0.61–1.05)	0.94 (0.74–1.18)
		Q3	0.88 (0.67–1.14)	0.60 (0.45–0.79)
		Q4	0.80 (0.61–1.05)	0.54 (0.40–0.72)
		Q5	0.76 (0.58–1.00)	0.58 (0.44–0.78)
	Model 1	Q1	1	1
		Q2	0.85 (0.64–1.13)	0.92 (0.72–1.17)
		Q3	1.01 (0.76–1.32)	0.61 (0.46–0.80)
		Q4	0.99 (0.74–1.30)	0.56 (0.41–0.76)
		Q5	1.04 (0.78–1.37)	0.67 (0.50–0.90)
The entire sample	Crude model	Q1	1 (Reference)	1 (Reference)
		Q2	0.73 (0.57–0.92)	0.77 (0.63–0.95)
		Q3	0.81 (0.64–1.02)	0.50 (0.39–0.63)
		Q4	0.81 (0.64–1.02)	0.43 (0.33–0.55)
		Q5	0.82 (0.65–1.03)	0.44 (0.34–0.56)
	Model 1	Q1	1 (Reference)	1 (Reference)
		Q2	0.81 (0.64–1.04)	0.80 (0.65–0.98)
		Q3	0.95 (0.75–1.21)	0.55 (0.43–0.70)
		Q4	1.01 (0.79–1.28)	0.49 (0.38–0.63)
		Q5	1.06 (0.84–1.35)	0.52 (0.40–0.66)

Anemia was defined as <13 g/dL for men and <12 g/dL for women; Model 1 includes age, race, education attainment, diabetes mellitus, and hypertension. TSH: thyroid stimulating hormone; FT4: free-thyroxin.

## DISCUSSION

A systematic review conducted by Floriani et al^8^ following 12,337 participants with available baseline TSH, FT4, and hemoglobin levels used multiple linear regression analysis to compare hemoglobin levels across TSH categories. They found that mean hemoglobin levels in patients with subclinical hypothyroidism were lower than in euthyroid individuals at baseline, but without clinical relevance because of the small difference. In our sample the number of participants with anemia was too small to permit analysis according to hypothyroidism or euthyroid participants, which restricted our analysis to the association of anemia with TSH and FT4 levels. We reported an association of increasing FT4 levels with lower odds of anemia (p<0.0001).

In the analysis of Wopereis et al^6^, TSH, FT4, and hemoglobin data of 42,162 participants, with 51.5% women, from 16 different cohorts were analyzed, of whom 459 (1.1%) had overt hypothyroidism, 2,930 (6.9%) had subclinical hypothyroidism, and 36,081 (85.6%) were euthyroid. Of those, 4,274 (10.1%) had anemia at baseline, 15.9% in the overt hypothyroid group and 11.6% in the subclinical hypothyroid group. They found no statistically significant differences between men and women when asserting the risk of developing anemia in any of the groups. For females: OR 1.79 (95%CI 1.30–2.47) in the overt hypothyroid group; OR 1.23 (95%CI 0.99–1.52) in the subclinical hypothyroid group. For males: OR 2.45 (95%CI 1.45–4.12) in the overt hypothyroid group; OR 1.27 (95%CI 1.03–1.57) in the subclinical hypothyroid group. These findings are similar to ours since we did not detect differences according to sex.

The analysis by Vliet et al^7^ using self-reported data on hypothyroidism of the UK Biobank observed higher odds of anemia for subclinical hypothyroidism, but no evidence of a causal relationship between subclinical hypothyroidism and anemia using euthyroid participants as the reference. It is important to note that the studies of Wopereis et al^6^ and Vliet et al^7^ analyzed the association of hypothyroidism and anemia while in our analysis we evaluated the association of anemia with TSH and FT4.

In the study of Puy et al^9^, 669 participants were divided in two groups for testing whether levothyroxine treatment leads to a rise in hemoglobin levels in people with subclinical hypothyroidism (placebo N=337 and levothyroxine N=332. Median age was 75 years for both groups). At baseline, 13.1% of the participants had anemia in the placebo group compared to 11.4% in the levothyroxine group (mean hemoglobin level was 13.8 g/dL for both groups). TSH levels had no impact on Hb levels after the observation period when patients underwent levothyroxine treatment in any of the subgroups, whether they were related to clinical risk factors such as smoking or alcohol status, or related to comorbidities, such as hypertension or diabetes mellitus.

A previous study in Korea^13^ showed an association similar to what was observed in our study. In a sample of 5,352 participants, there was an association of low levels of FT4 with higher frequency of anemia. This association was higher in women than men. In our sample, high levels of FT4 were associated with low odds of anemia both in men and women. Compared to the Korean analysis, in our study there were no differences in the frequency of anemia according to sex. Thyroid hormones have a very important role in hematopoiesis, especially for red blood cell production^14–17^, however, the mechanism to explain this association is not clearly understood^16–19^. The results of the study of Kim et al^13^ and the findings of the present analysis suggest that FT4 would be more important than TSH in the control of erythropoiesis.

The strengths of this study consists in the large number of participants in a highly mixed sample, setting it apart from previous studies. The multifactorial nature of the cohort allowed us to access a wide variety of variables for cross-sectional analysis. The study has several weaknesses. This is a cross-sectional analysis that can study association but no causality. The percentage of anemia diagnoses according to thyroid function in the total sample, only accounting for a total of 5.3% (N=703) of participants across all subgroups, limiting the statistical power of our analysis. We were also not able to analyze the association between hypothyroidism, hyperthyroidism and anemia because of the small number which restricted our analysis to TSH and FT4 levels with anemia. Such limitations must be taken into account when analyzing the results.

## CONCLUSION

The findings of this study show that higher levels of FT4 are associated with lower odds of anemia. No associations were found between TSH levels and anemia. In analysis, according to sex, no differences were found between men and women.

## Data Availability

The datasets generated and/or analyzed during the current study are available from the corresponding author upon reasonable request.
